# Prevention of Acoustic Trauma-Induced Hearing Loss by N-acetylcysteine Administration in Rabbits

**DOI:** 10.5812/atr.7839

**Published:** 2013-02-01

**Authors:** Masoud Motalebi Kashani, Hamidreza Saberi, Mitra Hannani

**Affiliations:** 1 Occupational Health Department, Health Faculty, Kashan University of Medical Sciences, Kashan, IR Iran; 2 Trauma Research Center, Kashan University of Medical Sciences, Kashan, IR Iran

**Keywords:** Acoustic Trauma, Hearing Loss, N-Acetylcysteine

## Abstract

**Background:**

Acoustic trauma is an injury to the hearing mechanisms in the inner ear due to excessive noise. This injury is the most prevalent cause of sensorineural hearing loss in humans, especially from occupational exposure. Previous studies have shown the essential role of free radical formation in the inner ear hearing loss caused by acoustic trauma.

**Objectives:**

This study was performed to determine the effect of N-acetylcysteine (NAC) administration for reducing acute acoustic trauma in rabbits.

**Materials and Methods:**

Twenty four rabbits were assigned to four groups including: control, noise plus saline, noise plus NAC administration (325 mg/kg body weight by intraperitoneal injection (IP), three days before exposure to noise and three days after noise exposure), and NAC alone. Auditory brain stem response (ABR) threshold was measured before exposure and one hour and 14 days after exposure.

**Results:**

The saline plus noise group had on average a 49 decibel (dB) temporary threshold shift (TTS) and 23.9 dB permanent threshold shift (PTS) at the studied frequencies, while rabbits in the NAC administration plus noise group had a 31.5 dB TTS and 10.7 dB PTS averaged across the frequencies.

**Conclusions:**

Administration of NAC can provide appropriate protection against acoustic trauma-induced hearing loss in rabbits at all studied frequencies.

## 1. Background 

Acoustic trauma is injury to the hearing mechanisms of the inner ear due to excessive noise (> 85 dB). Acoustic trauma is a prevalent cause of sensorinural hearing loss and it may be caused by an explosion close to the ear, gun shots, long–term exposure to loud noise (such as machinery noise) (1-3). In adults, acoustic trauma is the most important cause of noise induced hearing loss (NIHL). In total, 16% of disabling hearing losses in adults were attributed to noise exposure (4). Acoustic trauma after noise exposure is one of the most contemporary conditions in humans, especially for young people frequenting night clubs, musicians and industrial laborers (5). Acoustic trauma-induced hearing loss may be created by both direct mechanical trauma to the inner ear occurring during intense noise exposure, and as a result of oxidative stress in the cochlea hair cells. The noise, induced formation of reactive oxygen species (6) and reactive nitrogen species (RNS) in the cochlea, which are the main causes of oxidative stress. Previous studies have shown the essential role of free radical formation in the cochlea in hearing loss caused by acoustic trauma (6-9). These studies have shown that NIHL can be decreased by antioxidant agents acting as free radical scavengers. Many drugs have been used for the treatment and prevention of NIHL (10-13). Nowadays, pharmacological preventive measures for the control of NIHL can be important components for hearing loss prevention programs in workplaces. N-acetylcysteine (NAC) as an antioxidant agent and as a substrate for glutathione synthesis, with no noticeable side effects, has been used for the prevention and treatment of NIHL in many studies (2, 3, 14). These researches have shown that NAC can effectively reduce NIHL in animal models for long-term exposure to noise (chronic acoustic trauma) (12, 14, 15). However, there is no evidence to suggest that NAC is effective for protecting against acute acoustic trauma (excessive noise for short time exposure). Many studies have shown that NAC cannot reduce impulse noise trauma induced temporary threshold shift and loud music induced hearing loss (4, 5).

## 2. Objectives

This study was conducted to determine the effect of N-acetylcysteine (NAC) administration on reducing temporary and permanent injuries resulting from acute acoustic trauma (excessive noise for short time exposure) in rabbits.

## 3. Materials and Methods 

### 3.1. Experimental protocol

Twenty four healthy, male, adult white New Zealand rabbits (2200 – 2500 g body weight) were obtained from the Pasture Institute of Iran. The rabbits were accommodated in separate cages and maintained on a light–dark cycle (12 – 12 h) at 23-27°C, with free access to food. All procedures relating to the care, use and handling of the rabbits were conducted in accordance with the principles of the Helsinki Protocol. The rabbits were divided randomly into four groups (n = 6, each group) according to the type of experimental condition as follows: Group 1, control (no exposure to noise and no injection); Group 2, exposed to noise (120 dB octave band noise centered at 4 kHz for four hours) and received saline intraperitoneal injection (IP); Group 3, exposed to noise (as in Group 2) and received NAC 325 mg/kg body weight IP injection; and Group 4, not exposed to noise and received NAC (325 mg/kg body weight IP injection). NAC or saline was injected IP at a single dose (325 mg/kg body weight) once a day from three days before noise exposure to three days after noise exposure (6 days in total). NAC was administrated 325 mg/kg body weight because previous studies had shown that this dose was the most effective for the reduction of continuous noise induced hearing loss (2, 16). Groups 2 and 3 were exposed to octave band noise centered at 4 kHz, 120 dB SPL, for four hours. This level of noise can be enough to cause acoustic-trauma induced hearing loss in animal models (9, 14). The octave band noise was produced and amplified with a specific software program (Signal software) and released with a personal computer system using Cool Edit software (Adobe system in US) to speakers in an exposure chamber (the exposure chamber was designed as a reverberant field which could house six rabbits at a time). Noise levels were monitored continuously in the chamber by a precision sound level meter (Cel–460, UK).

### 3.2. Auditory Evaluation

Auditory brain stem response (ABR) was used to evaluate the rabbits’ auditory function. ABR is based on the recording of auditory evoked potentials and they are widely used to evaluate levels of acoustic trauma-induced hearing loss in animal models (12, 13, 17). ABR represents the neuroelectric activity in the brainstem auditory pathways and can assay cochlear function. The resulting recording form a series of vertex positive waves, which are evaluated I through V. These waves are labeled with roman numerals. ABR threshold was described as the lowest stimulus intensity that creates a reproducible ABR wave form peak III or IV (18). ABR threshold and latency time of wave V are the two important criteria for evaluating function auditory levels (19). Many studies showed significant correlations between ABR thresholds and behavioral thresholds in animal models (20-22). In this study, ABR thresholds and latency time wave V at 1, 2, 4 and 8 kHz were measured in all experimental groups (blindly) at three time points: Prior to the experiment (before noise exposure and saline or NAC injection) to determine the baseline ABR threshold and latency time wave V, one hour and 14 days after noise exposure. The tests were done at 1, 2, 4 and 8 kHz tone bursts stimuli (1 ms Blackman rise/fall, 15 ms duration, and alternating polarity) produced using Interacoustics Eclipse EP25. Stimuli were delivered through a computer-controlled insert phone located in the rabbit’s ear canal. An active needle electrode was placed subcutaneously below the test ear. Reference electrode and ground electrode were placed at the vertex and the other ear, respectively. The rabbit’s ABR threshold at studied frequencies for a specific time was defined as the average ABR threshold of the left and right ear. The difference between baseline ABR threshold (before noise exposure) and ABR threshold one hour after noise exposure was defined as the temporary threshold shift (TTS). Permanent threshold shift (PTS) was considered as the difference between baseline ABR threshold and ABR threshold 14 days after noise exposure at each frequency. Before auditory evaluation, all rabbits were anesthetized with xylazine (10 mg/kg bodyweight) and ketamine (40 mg/kg bodyweight) mixture by intramuscular injection (IM). External ear canals were examined to ensure that the canal was free of wax.

### 3.3. Statistical Analysis

The Kolmogorov-Smirnov test (K-S test) was used to evaluate the normality of data in all groups. One-way analysis of variance (ANOVA) was used to compare ABR threshold shifts among groups at the study frequencies. Tukey’s test was applied as a post-hoc test for multiple comparisons among the groups. Latency time of wave V before and after noise exposure was compared using a paired t-test at each frequency in all groups. A P value smaller than 0.05 was considered significant.

## 4. Results

**Figure 1:**
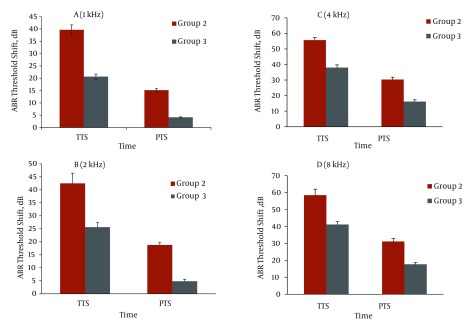
ABR Threshold Shifts, One Hour After Exposure to Noise (TTS) and 14 Days After Exposure to Noise (PTS) in Groups 2 and 3 at 1 kHz (A), 2 kHz (B), 4 kHz (C) and 8 kHz (D) ^a^ Abbreviations: ABR, auditory brain stem response; PTS, permanent threshold shift; TTS, temporary threshold shift ^b^ Bars show means; Error bars show ± 1.0 S.D; Group 2, noise exposure + saline IP injection; Group 3, noise exposure + NAC 325 mg/kg IP injection.

Both ABR threshold and latency time of wave V before exposure (baseline) at each frequency were equal in all the experimental groups. The latency time wave V at 8 and 4 kHZ were significantly lower than 2 and 1 kHZ in all groups. The latency time wave V before exposure and one hour after exposure at each frequency in the experimental groups are represented in Table 1. Statistical comparisons showed that the latency time one hour after exposure in groups 1 and 4 were not significantly different from latency time before exposure. In groups 2 and 3 latency time one hour after exposure was significantly more than baseline latency time at all frequencies (P < 0.001). In Table 2, baseline latency time and latency time 14 days after exposure are represented. According to Table 2, latency time 14 days after exposure was significantly greater than baseline at all frequencies in group 2, but in group 3 the latency time 14 days after exposure at 8 kHz was more than baseline (P < 0.001). There was no significant difference between latency time 14 days after exposure and baseline at each frequency in groups 1 and 4. ABR threshold shift at one hour after exposure (temporary threshold shift, TTS) and ABR threshold shift at 14 days post exposure (permanent threshold shift, PTS) at each frequency in the experimental groups 2 and 3 are shown in Figure 1. ABR temporary threshold shift and ABR permanent threshold shift of group 1 (control) and group 4 (NAC administration) were approximately 0. The saline + noise group (group 2) had a 49 dB TTS and 23.9 dB PTS averaged across frequencies of 1 - 8 kHz, while rabbits receiving NAC plus noise (group 3) had a 31.5 dB TTS and 10.7 dB PTS averaged across the frequencies. The ABR threshold shift (TTS and PTS) of groups 2 and 3 at 4 and 8 kHz were significantly higher than at 1 and 2 kHz (P < 0.01). The acoustic trauma induced ABR threshold shifts at all studied frequencies, and were significantly decreased in rabbits which were exposed to noise and received NAC (group 3) compared to animals which were exposed to noise and received saline (group 2) at one hour and 14 days post-exposure to acoustic trauma (P < 0.01). This reduction at 4 and 8 kHz was significantly smaller than at 1 and 2 kHz (P < 0.01).

**Table 1:** Mean and Standard Deviation of Latency Time Wave V in Milliseconds (ABR Test), Before and One Hour After Exposure in Experimental Groups

Frequency, kHz	Latency Time Wave V, ms		P value
	Before Exposure, Baseline, Mean ± SD	One Hour After Exposure, Mean ± SD	
**Group 1 (Control)**			
**1**	4.77 ± 0.020	4.77 ± 0.022	1
**2**	4.74 ± 0.019	4.74 ± 0.019	1
**4**	4.71 ± 0.016	4.71 ± 0.019	1
**8**	4.67 ± 0.014	4.67 ± 0.016	1
**Group 2 (Noise + Saline)**			
**1**	4.77 ± 0.020	5.30 ± 0.040	< 0.001
**2**	4.74 ± 0.019	5.32 ± 0.059	< 0.001
**4**	4.71 ± 0.016	5.74 ± 0.04	< 0.001
**8**	4.67 ± 0.014	5.49 ± 0.051	< 0.001
**Group 3 (Noise + NAC a)**			
**1**	4.79 ± 0.027	5.03 ± 0.021	< 0.001
**2**	4.76 ± 0.030	5.08 ± 0.015	< 0.001
**4**	4.74 ± 0.034	5.23 ± 0.021	< 0.001
**8**	4.68 ± 0.035	5.27 ± 0.016	< 0.001
**Group 4 (NAC)**			
**1**	4.77 ± 0.038	4.77 ± 0.037	0.363
**2**	4.74 ± 0.030	4.75 ± 0.031	0.175
**4**	4.71 ± 0.024	4.72 ± 0.024	0.175
**8**	4.67 ± 0.028	4.67 ± 0.027	0.175

^a^Abbreviation: NAC, N-acetylcysteine

**Table 2:** Mean and Standard Deviation of Latency Time Wave V in Milliseconds (ABR Test), Before and 14 Days After Exposure in Experimental Groups.

Frequency, kHz	Latency Time Wave V, ms		P value
	Before Exposure (Baseline), Mean ± SD	14 Days After Exposure, Mean ± SD	
**Group 1 (Control)**			
**1**	4.77 ± 0.020	4.77 ± 0.022	1.175
**2**	4.74 ± 0.019	4.74 ± 0.020	1.175
**4**	4.71 ± 0.016	4.71 ± 0.016	1.363
**8**	4.67 ± 0.014	4.67 ± 0.014	1.175
**Group 2 (Noise + Saline)**			
**1**	4.77 ± 0.020	5.30 ± 0.014	< 0.001
**2**	4.74 ± 0.019	5.32 ± 0.015	< 0.001
**4**	4.71 ± 0.016	5.74 ± 0.29	< 0.001
**8**	4.67 ± 0.014	5.49 ± 0.035	< 0.001
**Group 3 (Noise + NAC a)**			
**1**	4.79 ± 0.027	5.03 ± 0.027	1
**2**	4.76 ± 0.030	5.08 ± 0.030	1
**4**	4.74 ± 0.034	5.23 ± 0.076	0.087
**8**	4.68 ± 0.035	5.27 ± 0.017	< 0.001
**Group 4 (NAC a)**			
**1**	4.77 ± 0.038	4.77 ± 0.036	0.175
**2**	4.74 ± 0.030	4.75 ± 0.033	0.093
**4**	4.71 ± 0.024	4.72 ± 0.026	0.081
**8**	4.67 ± 0.028	4.67 ± 0.034	0.126

^a^Abbreviation: NAC, N-acetylcysteine

## 5. Discussion

The purpose of the study was to investigate the preventive effects of NAC administration on acoustic trauma induced hearing loss in rabbits. Our results showed that when NAC was administered three days before acoustic trauma and three days after exposure to noise (325 mg/kg body weight, IP injection), an appropriate prevention was provided against acute acoustic trauma induced temporary and permanent hearing loss in rabbits. Acoustic trauma can cause reactive oxygen species (ROS) formation in the cochlea as a normal cellular byproduct. In a normal status, various natural antioxidant defense mechanisms may prevent these damaging free radicals from causing any permanent injury, but when acute acoustic trauma occurs, normal antioxidant defense mechanisms are no longer maintained and hair cells in the cochlea may undergo a non-recoverable injury leading to permanent hearing loss. In this situation, our results suggest that temporary and permanent hearing loss may be prevented or reduced by the administration of an antioxidant agent (NAC). This improvement may be due to glutathione (GSH) synthesis by NAC. GSH may also be able to recover the effects of glutamate exciototoxicity as well as protect cochlear mitochondria from free radical formation (14, 15). NAC is known to be an effective radical scavenger for hydroxyl radical, hydrogen peroxide and hypochlorous acid. By increasing GSH production in cell, there may also be less oxidative stress in the cochlea (23). Acute acoustic trauma can damage the cochlea through the formation of ROS and RNS, lipid peroxides and produce a reduction of cochlear GSH and other cellular antioxidants. NAC is an effective supplier of inherent antioxidants (GSH) and it can also reduce ROS and RNS (24). NAC as a GSH precursor and antioxidant can prevent cochlear damage through free radical scavenging and providing a substrate for cochlear GSH synthesis. Administration of NAC was started three days before the acoustic trauma to prepare a stable and higher concentration of GSH in the cochlea at the beginning of the acoustic trauma. Moreover, NAC was administrated three days after noise exposure because there is evidence for delayed free radical formation, 7 - 10 days following noise exposure (6). According to the results of the present study, permanent ABR threshold shifts in group 2 (noise + saline) are more significant than in group 3 (noise + NAC) at each frequency. This means that NAC can ameliorate acoustic trauma induced permanent hearing loss at all frequencies. The results are in agreement with previous researches (2, 12, 25). Statistical comparisons showed that NAC attenuated temporary and permanent ABR threshold shifts more significantly at 1 and 2 kHz than at 4 and 8 kHz, while noise could cause more temporary and permanent ABR threshold shifts at 4 and 8 kHz than at 1 and 2 kHz. This finding is consistent with those of the Ohinata et al. and Yamasoba et al. studies showing that antioxidant agents were more effective at frequencies further from the frequencies with greater threshold shifts (26, 27). This may be due to other factors, in addition to ROS formation. Results from this study indicate that NAC temporarily attenuated ABR threshold shifts at all frequencies. These findings are in disagreement with those of Karmer et al. and Duan et al. studies, in that, they found no correlation between NAC administration and TTS attenuation (2, 3). This difference may be due to a difference between noise levels, animal model and dose of NAC administered. Although NAC administration could ameliorate acoustic trauma induced temporary threshold shift, however, according to the values presented in Table 1, it could not reduce TTS to baseline and according to the values presented in Table 2, it was able to reduce PTS to baseline at 1, 2 and 4 kHz, but it could not return hearing levels to baseline at 8 kHz. This means that NAC can attenuate acoustic trauma induced TTS and PTS, but it is not able to restore the hearing level to the initial condition. This finding is consistent with previous researches about protective effects of antioxidant agents against noise induced hearing loss (6, 8, 9, 26). NAC, a thiol containing amino acid derivative is used throughout the world as a nutritional supplement and also as a drug which has been approved by the Food and Drug Administration (FDA), could be a suitable candidate for the prevention and treatment of acoustic-trauma induced hearing loss in human. We recommend future systematic human clinical trials to demonstrate the potential of NAC administration for the prevention of acoustic trauma-induced hearing loss in humans. Administration of NAC (325 mg/kg body weight) by intraperitoneal injection, three days before exposure to noise and three days post-exposure to noise, can provide appropriate protection against acoustic trauma-induced hearing loss.
